# Taxonomic affiliation influences the selection of medicinal plants among people from semi-arid and humid regions—a proposition for the evaluation of utilitarian equivalence in Northeast Brazil

**DOI:** 10.7717/peerj.9664

**Published:** 2020-08-04

**Authors:** Rafael Reinaldo, Ulysses Albuquerque, Patrícia Medeiros

**Affiliations:** 1 Departamento de Biologia, Universidade Federal Rural de Pernambuco, Recife, Pernambuco, Brazil; 2 Centro de Biociências, Universidade Federal de Pernambuco, Recife, Pernambuco, Brazil; 3 Campus de Engenharias e Ciencias Agrárias, Universidade Federal de Alagoas, Rio Largo, Alagoas, Brazil

**Keywords:** Ethnopharmacology, Traditional knowledge, Use patterns, Selection criteria, Ethnobotany

## Abstract

**Background:**

This study sought to investigate the occurrence of taxonomic patterns between semi-arid and humid regions, verifying how the taxonomic affiliation can influence the selection of plants for medicinal purposes and act as a selection criterion.

**Methods:**

The relationship between the taxonomic affiliation and the selection of medicinal plants with four different communities was analyzed; two of them associated with a seasonally dry tropical forest and the other two associated with a tropical rain forest. We used the Utilitarian Equivalence Model (transposing the concept of ecological equivalence, proposed by Odum, for ethnobotany) to test the hypothesis that species that have the same taxonomic affiliation tend to have the same therapeutic applications in different environments (utilitarian equivalence). In addition, we used the Utilitarian Redundancy Model to verify whether, within the same medical system, plants of the same taxonomic affiliation tend to be redundant (treating the same diseases).

**Results:**

We found that a pair of plants of the same genus were 9.25 times more likely to be equivalent than a different genus pair (OR = 9.25, CI [1.68–51.02], *p* < 0.05). When we analyzed the species used by the same population, the chances of a pair having similar therapeutic uses (utilitarian redundancy) increased when they were species of the same family (OR = 1.94, CI [1.06−3.53]; *p* < 0.05).

**Conclusions:**

These findings confirm the hypothesis that there is an influence of taxonomic affiliation, in terms of genera and family, on the selection of medicinal plants in semi-arid and humid areas in Northeast Brazil. In addition, our Utilitarian Equivalence Model can be an important tool in the search for more common selection criteria, in order to identify the shared characteristics among the equivalent pairs and consequently the main types of perceptions or stimuli that led to the inclusion of such species in local pharmacopoeias.

## Introduction

Local plant-based medical systems, which include the whole set of knowledge, beliefs and practices to deal with diseases, with plants as a main material constituent, have been studied as a model to better understand the relationship between people and natural resources (Moerman, 1991; Brown et al., 2011; Saslis-Lagoudakis et al., 2012; Ferreira Júnior et al., 2015). Considering that human behavior on plant use depends on biological, sociocultural and environmental factors, an increasing concern among ethnobotanists is to clarify how these factors act and interact generating use patterns. In this sense, many advances in ethnobotany included the development of techniques, methods and hypotheses based on the theoretical framework of ecology, adapting ecological models to the scenario of complex interactions between biology and culture (Albuquerque & Oliveira, 2007).

The Utilitarian Redundancy Model (URM), for example, started from ecological redundancy and was originally created with the purpose of explaining how the existence of botanical species with high overlapping uses can contribute to the maintenance and resilience of a given Local Medical System (Albuquerque & Oliveira, 2007; Nascimento et al., 2015). The main assumption of URM, already demonstrated in empirical studies, is that redundant species assure the functions of the system in the absence of other species that have the same function (Santoro, 2014).

Another important proposition is the ecological apparency hypothesis that, in the context of ethnobotany, explains the high amount of herbaceous plants in local pharmacopoeias because of the existence of strongly bioactive qualitative compounds (Phillips & Gentry, 1993). These and other recent propositions differ from the old quantitative approaches because they assume that socioecological systems and conventional ecosystems are governed by some variables that are common to them, especially the influence of the environment, as some empirical studies have shown (Gonçalves, Albuquerque & Medeiros, 2016). Despite notable theoretical advances, there are still gaps, especially regarding the similarities and differences between medical systems.

The Local Medical Systems of different peoples around the world have dynamics and structures that, directly or indirectly, reflect the different cultural traits of a given human population (Ankli, Sticher & Heinrich, 1999). In this sense, people with distinct cultural origins could present significant differences in the mode of selection of medicinal plants (Ankli, Sticher & Heinrich, 1999). However, there is also evidence that two distinct human cultures, living far apart but inserted in similar environments, tend to select medicinal plants in a similar way, as there would be a strong positive correlation between the medicinal flora and the floristic environment (Saslis-Lagoudakis et al., 2014). In other words, similarities as to the diversity of plant species available may lead to similarities in medicinal systems (Saslis-Lagoudakis et al., 2014).

Given this duality between culture and the environment, some studies on the medicinal flora of different regions of the world have been demonstrating certain patterns of use. They are suggesting that, even when medicinal floras are essentially composed of different species, plants used for the same purposes tend to share common traits, such as the same taxonomic group (Leonti et al., 2003, 2009; Saslis-Lagoudakis et al., 2012).

One of the most interesting models to understand how environmental and cultural aspects can guide local systems is the study of the therapeutic use of plants in various human groups (Ferreira-Junior & Albuquerque, 2018). In addition, a good scenario to study the behavior of medicinal plants is the study of essentially different environments, comparing, for example, humid environments vs. arid environments (Medeiros, Ladio & Albuquerque, 2015), considering that, if certain use behaviors persist even among areas that suffer different environmental pressures, these can be a pattern of behavior. Although the present study follows a similar approach to those discussed above, it is proposed to use the concept of utilitarian equivalence, a new framework, based on Odum (1971) ecological equivalence model. The Utilitarian Equivalence Model aims to better understand cases of overlap uses between medicinal floras from different regions.

### Ecological equivalence

This study considers the concept of ecological niche, one of the central elements of ecology. In the words of Begon, Townsend & Harper (2006) ecological niche corresponds to “the ways in which tolerances and requirements interact to define the conditions and resources needed by an individual or species to practice their way of life”. In other words, it is the multidimensional role of a species or an individual in the functioning of a given ecosystem (Hutchinson, 1957). In this sense, it was suggested by Odum (1971) that “organisms that occupy the same ecological niches or similar ecological niches in different geographic regions…” could be called ecological equivalents. Odum (1971) also points out that “species that occupy equivalent niches tend to be closely related from a taxonomic point of view, although often they are not in regions that are very separate or isolated from one another”.

For Odum (1971), distinct biogeographic regions presuppose “species composition of quite different communities”. Where there are physically similar habitats, similar ecosystems develop (Odum, 1971). Thus, “functional niches are occupied by equivalent biological groups, regardless of the composition of the fauna and flora of the region” (Odum, 1971). From this perspective, we can consider, for example, that a prairie ecosystem will occur in any region of the world that has a prairie-friendly climate, with local native species being organized to occupy available niches (Odum, 1971).

Odum (1971) highlighted ecological equivalents in three trophic niches of four coastal regions and presented similar herbivorous littorina belonging to the same genus, thus reinforcing the idea that ecological equivalence may undergo taxonomic influence. Some empirical evidence on the subject can be found in Fišer et al. (2015). When considering ideas similar to those of Odum, Fišer et al. (2015) evaluated the overlap level of niches between cryptic species of crustaceans of the genus Niphargus and their results suggest that, on a regional scale, the species play equivalent ecological roles, which in turn may be related to a still recent speciation process (Fišer et al., 2015).

In light of the mentioned above, the equivalence model can help to examine if, in a similar way, the occurrence of species in different regions with high overlapping of medicinal use is due to similarities between them, thus allowing to elucidate aspects of the selection of medicinal species.

### Utilitarian equivalence

The present study transposes for ethnobotany a concept analogous to that of ecological equivalence, hereafter called utilitarian equivalence, and uses it as an analytical tool in the search for common criteria for the selection of medicinal plants by people in semi-arid and in humid regions in the Northeast of Brazil. This proposition is based on the understanding that access to equivalent medicinal species provides the ideal scenario to seek common selection criteria, in order to identify the shared characteristics among the equivalent pairs and consequently the main types of perceptions or stimuli, which led to the inclusion of such species in local pharmacopoeias.

The term utilitarian equivalence, here proposed, indicates species that are used for the same purposes or similar purposes in different socioecological systems. In this study, we used local medical systems as a model. However, the term is not restricted only to the therapeutic use of plants, and this approach can be directed to the diverse applications that any biological resources receive from human groups.

Our model is based on the assumptions that: (a) utilitarian equivalence, understood as the high overlap of use between two species in distinct socioecological systems, is relative and not absolute, since, in the absence of intrinsically identical plant species or culturally equal peoples, the medicinal uses are not necessarily identical, but rather similar; (b) equivalence is due to two groups of complementary variables, cultural traits and environmental factors; (c) the evolutionary events that led to utilitarian equivalence may be associated with the similarity between intrinsic characteristics of useful species.

### Is utilitarian equivalence the same as ethnobotanical convergence?

The concept of utilitarian equivalence proposed here is different from the concept of ethnobotanical convergence presented by Garnatje, Peñulas & Vallès (2017). Although both deal with different species used for the same purposes, the first has as its central element the overlap of uses between species of different medical systems. The concept of “ethnobotanical convergence” is different from that of utilitarian equivalence, especially since it is restricted to “similar uses for plants included in the same node of a phylogeny” (Garnatje, Peñulas & Vallès, 2017). Thus, a high overlap of medicinal uses between phylogenetically distant plants, such as a fern in Africa and an angiosperm in North America, can be considered a case of utilitarian equivalence, but not of “ethnobotanical convergence”. On the other hand, if the ethnobotanical convergence is established between species of different Local Medical Systems, there is also utilitarian equivalence.

The concept of utilitarian equivalence is also different from the concept of ethnobotanical convergence from Hawkins & Teixidor-Toneu (2017). The authors reviewed the idea of Garnatje, Peñulas & Vallès (2017) and proposed an adjustment that considered the accepted meaning of the term convergence, indicating that ethnobotanical convergence should be only used in cases where the independent discovery of the medicinal potential is evident. Therefore, it does not apply to cases of overlapping uses that are a consequence of the diffusion of knowledge among different peoples (Hawkins & Teixidor-Toneu, 2017). This distinction is important because one of the main applications of studying ethnobotanical convergence is bioprospecting. Therefore, closely related plants that are used by different populations for the same end may have similar chemical characteristics that justify such convergence (Hawkins & Teixidor-Toneu, 2017).

In the case of utilitarian equivalence, it can be used in the search for environmental or cultural selection criteria. Therefore, taxonomy is only one possible driver of utilitarian equivalence, but other aspects such as a plant’s habit, flavor or availability may play important roles in such process. Furthermore, since our concept does not require independence of the studied human populations, any taxonomic patterns found may be due to (1) shared chemical characteristics of closely related plants, or (2) cultural diffusion leading to attribution of similar uses to closely related plants because of their morphological similarity.

Finally, convergence is established when closely related plants share individual uses (e.g., a single therapeutic indications or categories of conditions). As for utilitarian equivalence, it is only established when two species have a substantial overlap of uses.

Following this perspective, the present study tried to test the following hypotheses:

1. In distinct medical systems, taxonomically close species tend to present utilitarian equivalence. It was expected that pairs of species with the same taxonomic affiliation, in terms of genus and/or family, would be more likely to be utility equivalents than taxonomically distant plants.
2. Within a medical system, taxonomically close species tend to present utilitarian redundancy.

It was expected that pairs of species having the same taxonomy in terms of genus and/or family would be more likely to be utility redundant than taxonomically distant plants.

## Materials and Methods

### Study area

Within the purpose to verify if certain use behaviors persist even between areas with different floristic compositions and social-ecological contexts, also minimizing the probability of results being skewed by knowledge transmission, it was decided to test the utilitarian equivalence model among communities inserted in two areas from different ecosystems and essentially distinct and distant from each other. In this sense, the study was conducted in four rural communities, two neighboring communities in an area in a tropical rainforest (TR), in a hot and humid climate region, and two neighboring communities in a seasonally dry tropical forest area (SDTF), in a hot and dry climate region with semi-arid regime, all located in the Northeast of Brazil.

We chose to compare different ecosystems because we intended to evaluate if, even under completely different social-ecological conditions, some taxonomic patterns on plant use could emerge. Therefore, these communities may not be seen as replicates, but rather as case-studies. Additionally, our choice for including more than one community in a single region was also not related to the production of replicates. We did not compare neighboring communities, as our intention was to consider them as a continuum. Thus, we adopted a meta-community approach, since, for both cases, we chose neighboring communities.

### Tropical rainforest

The Murici Ecological Station (ESEC-Murici), a large forest fragment located in the municipality of Murici-Alagoas—Northeast of Brazil, 60 km from the state capital, Maceió, represented the Tropical Rainforest Area, locally named the Atlantic Forest. The surrounding environment is basically composed of areas of sugar cane cultivation, although there are also other interface situations such as cattle raising and eucalyptus plantation. The vegetation is considered Dense Ombrophylous Forest and has an area of 6.116 ha (IBAMA, 2006). The region presents a humid to subhumid tropical climate, with two well-defined seasons: dry summer, which runs from September to March and rainy winter, which runs from April to August (IBAMA, 2006). The climate of the region is type As, according to Köppen classification, with rainfall totals ranging from 800 to 1,800 mm and average temperature of 25 °C (IBAMA, 2006). Near the forest, there is a predominantly agricultural human population that exploits forest resources for survival (IBAMA, 2006).

The rural communities located in the Atlantic Forest were the Dom Helder Câmara settlement and the Che Guevara settlement, both belonging to the municipality of Murici/Alagoas (S 9°18′26″ W 35°55′55″). A total of 204 people live in the settlement Dom Hélder Câmara, while the Settlement Che Guevara has 220 residents. Local populations are mainly made up of former landless laborers whose main occupation was planting sugarcane on rural properties in surrounding municipalities, and settled in the settlements around the year 2000, when the areas were made available for agrarian reform (Cavalcanti & Barros, 2006). Although there is a low level of education among adults, each of the communities has a municipal public school offering basic education. In addition, there is regular school transportation for students who attend high school in public schools in the urban area. Hospitals and health posts are restricted to the urban area (SEPLAG/AL, 2012), distant approximately 13 km from the community. Even with the possibility of access to health care by school transports and similar means, it is possible to note that part of the local population remains resistant to medical consultations and exhibits a rich traditional medicinal repertoire based mainly on plant species.

As for religious aspects, the majority of the population of the settlements Dom Helder Câmara and Che Guevara is adept to Christian Religious Doctrines. However, in both communities it is also possible to observe healing practices originating from religions of African and indigenous matrices oriented towards magic-religious use involving the use of plants.

### Seasonally dry tropical forest

The area of the seasonally dry tropical rainforest selected, locally known as the Caatinga, was the Catimbau National Park (PARNA Catimbau), an Integral Protection Conservation Unit, created by a decree on December 13th, 2002. The park has about 62,000 ha and is located in the municipalities of Ibirimirim, Tupanatinga and Buíque in the state of Pernambuco, 295 km from Recife, the state capital. Although it is a permanent conservation area, forest areas are not continuous, with vegetation patches dominated by shrubs or herbaceous plants, which is probably related to a degradation process caused by chronic disturbances due to antrophic activities (Ribeiro et al., 2015) The park is located in the transition zone between the agreste and the sertão, and presents a Bsh climate according to the classification of Köppen (Alvares et al., 2013), with transition to tropical humid, with rainfall ranging from 650 to 1,100 mm.

The two communities in the seasonally dry tropical rainforest region were Igrejinha and Batinga, both belonging to the municipality of Buíque—Pernambuco—Northeast of Brazil (S 08°37′23″ W 37°09′21″). Approximately 171 and 91 people inhabit the communities of Igreginha and Batinga, respectively. The population of both communities carry out subsistence agriculture and has a close dependance on plant and animal species native to the region, both for food and for medicinal purposes. The vast majority of the population did not attend schools, especially the older population. However, both communities have primary schools. Most of the inhabitants have always lived in the communities where they now live, having a long and close relationship with the native vegetation.

The Igrejinha community has only one church that is Protestant, and just as in the communities described above, most people are adept of Christian Religious Doctrines. On the other hand, the inhabitants of the Batinga community affirm they are descendants of indigenous peoples and perform various healing and magico-religious rituals that rely on the use of plant species. The nearest Hospitals and health posts are restricted to Catimbau Village, an urban area that is about 20 km away. Access is possible from private vans who make the transfer at certain times of the day. Access to health services is difficult and part of the population prefers to treat themselves with medicinal plants.

The criteria for choosing the communities studied included: (1) rural communities; (2) have a minimum distance of 10 km in the urban environment; (3) are located close to native vegetation, at a distance of 1 km from the forest environment.

### Ethnobotanical survey

The ethnobotanical survey took place between January 2017 and January 2018 (on average 15 days a month) and included the selection of informants and semi-structured interviews (Supplemental File 1). Given the need to obtain a reliable sample of the medicinal repertoire of the communities, we chose to carry out a non-probabilistic selection of informants, recruiting only locally legitimated people and recognized as knowledgeable about medicinal plants (Table 1). In this stage, the Snowball method (Bailey, 1994) was used, which consisted of asking a local medicine specialist to indicate the specialists they knew. The same process was performed with the others until the indicated names repeated. Participants younger than 18 years were not included.

**Table 1:** Distribution of local experts interviewed by community and by sex.

Ecosystem	Community	Men	Women	Total
Atlantic Forest	Ass. D. Helder Câm	15	11	26
	Ass. Che Guevara	11	2	13
Caatinga	Igrejinha	26	34	59
	Batinga	13	16	29
		65	63	127

With each informant, a semi-structured interview was carried out in association with the free-listing method (Albuquerque et al., 2014) in which the interviewees were asked about the medicinal plants they knew, thus generating an initial list with the names of the plants used. For each ethnospecies (ethnobiological taxonomic category based on local communities’ classifications) mentioned, the following question was asked: Is this plant important in the treatment of health problems? The information obtained was recorded field notebooks and forms.

### Collection of botanical material and taxonomic identification

For the taxonomic identification of the mentioned species and validation of the popular names attributed to the respective plants, guided tours were performed in forest areas commonly frequented to collect the medicinal plants. In the guided tour method, at least one member of the community with extensive knowledge of the local flora and the study area is invited to identify within the forests the listed ethnoespecies (Albuquerque, Lucena & Lins-Neto, 2014). Two informants from each community were selected for each collection, using as criteria the highest amounts of plants cited by each informant.

The identification of the species collected was performed by the Instituto Agronômico de Pernambuco (IPA), and was based on the APG IV classification system (Chase et al., 2016). The control material was deposited in the IPA Herbarium. One point to be highlighted is that not all the ethnospecies mentioned could be collected and/or identified, mainly due to seasonality or the rarity/absence of the species in the region. When this happened, the species (in this case, ethnospecies) was excluded from the analyzes.

### Ethical and legal aspects

An authorization for scientific activities in the Catimbau National Park area (PARNA Catimbau) was requested from Instituto Chico Mendes de Conservação da Biodiversidade (ICMBio/SISBIO), an agency linked to the Brazilian Ministry of the Environment (MMA), for the collection of botanical material (authorization number 67801-1). In addition, the present study was approved by the Human Research Ethics Committee (CEP) of the Universidade Federal de Pernambuco under the authorization number 30919514.9.0000.5207, following resolution 466/2012 of the National Health Council in Brazil. In accordance with this resolution, the informants who agreed to participate in the study were asked to sign the Informed Consent Form, through which they authorized the application of forms and interviews, as well as the publication of the data obtained.

### Data processing

The data obtained from the semi-structured interviews were filtered (Supplemental File 2) by inclusion and exclusion criteria described below.

### Initial selection of therapeutic and species indications: inclusion and exclusion criteria

This study considered only the therapeutic indications cited by two or more people in a given region. For example, if the plant “A” of the Seasonally Dry Tropical Forest was assigned only “analgesic” and “cancer treatment” uses and this information was cited by a single informant, this information was disregarded and consequently plant A was excluded from the analyzes. Similarly, if in a given region none of the information on how to treat a particular disease was shared by two or more informants, the disease was excluded from the study. This procedure was designed to prevent idiosyncratic information from biasing results and conclusions.

### Secondary selection of species and therapeutic indications: inclusion and exclusion criteria

This study was conducted under an etic approach, that is guided by the researcher’s perspective. In this context, only the mentions of diseases and symptoms accepted within the scope of conventional medicine were analyzed. After initial selection of therapeutic indications and species, only indications that remained in both the Seasonally Dry Tropical Forest and the Tropical Rain Forest were included. This consequently led to the exclusion of plant species that dealt only with the disregarded diseases.

By accounting for the information that fit the inclusion criteria defined herein, we had 27 therapeutic indications and 64 plant species, seven of which are listed in both the Atlantic Forest and the Caatinga (Table 2). The study considered both native and exotic species.

**Table 2:** Taxonomic affiliation, therapeutic indications and popular names of the 64 species obtained from the local experts of each phytophysiognomic region (Seasonally Dry Tropical Forest and Tropical Rainforest).

Family	Genus	Medicinal plants	Common name	Origin	Region*	Part of the plant used	Therapeutic indications	Herbarium voucher
Adoxaceae	*Sambucus*	*Sambucus nigra* L.	Sabugueira	Exotic	SDTF	Flower	Flu & colds & Cough	HST22162
					TR	Flower & Leaves	Colds & flu	Sterile material
Amaranthaceae	*Dysphania*	*Dysphania ambrosioides* (L.) Mosyakin & Clemants	Mastruz	Exotic	SDTF	Entire plant or Roots	Expectorant, Injury, Bone fracture, Flu & colds, Cough, Worms & Stomach problems	IPA91613
					TR	Leaves	Expectorant, Flu & colds, Cough & Worms	IPA91714
Anacardiaceae	*Anacardium*	*Anacardium occidentale* L.	Cajueiro roxo	Native	SDTF	Bark & Leaves	Toothache, Injury, Stomach problems & Inflammation in general	Sterile material
					TR	Bark & fruit	Toothache, Injury, Gynecological & Problem	Sterile material
	*Myracrodruon*	*Myracrodruon urundeuva* Allemão	Aroeira	Native	SDTF	Bark, stem, flower & Leaves	Pain in general, Acariasis & other infestations, Injury, Stomach problems, Indigestion, Inflammation in general & Cough	Sterile material
	*Schinopsis*	*Schinopsis brasiliensis* Engl.	Baraúna	Native	SDTF	Bark, stem, Leaves, sap & resin	Headache, Flu & colds	Sterile material
	*Schinus*	*Schinus terebinthifolia* var. acutifolia Engl.	Aroeira	Native	TR	Bark, Leaves	Injury, General inflammation & Gynecological problem	Sterile material
	*Spondias*	*Spondias tuberosa* L.	Umbuzeiro	Native	SDTF	Bark & Leaves	Calming, Diarrhea & Insomnia	Sterile material
	*Tapirira*	*Tapirira guianensis* Aubl.	Cupiuba	Native	TR	Sap & Resin	Injury	Sterile material
Annonaceae	*Xylopia*	*Xylopia frutescens* aubl.	Imbira Vermelha	Native	TR	Seeds.	Pain in general	Sterile material
Arecaceae	*Syagrus*	*Syagrus coronata* (Mart.) Becc.	Coco Ouricuri	Native	SDTF	Roots	Airways inflammation & Spine problems	Sterile material
Asphodelaceae	*Aloe*	*Aloe vera* (L.) Burm. f.	Babosa	Exotic	SDTF	Bark, Leaves & roots	Expectorant, injury, stomach problems, flu & colds, cough & worms	Sterile material
Asteraceae	*Acanthospermum*	*Acanthospermum hispidum* DC.	Federação	Native	SDTF	Leaves & roots	Expectorant, cough, flu & colds	IPA91626
					TR	Flower & roots	Cough	Sterile material
Bignoniaceae	*Handroantus*	*Handroantus impetiginosus* (Mart.ex DC.) Mattos	Pau D’arco Roxo	Native	SDTF	Bark	Stomach problems	Sterile material
					TR	Bark	Wounds	Sterile material
Bromeliaceae	*Neoglaziovia*	*Neoglaziovia variegata* (Arruda) Mez	Caruá	Native	SDTF	Roots	Spine problems	IPA91701
Burseraceae	*Commiphora*	*Commiphora leptophloeos* (Mart.) J.B.Gillett	Imburana de cambão	Native	SDTF	Bark & Leaves	Diarrhea, Injury, Hypertension & Cough	IPA91663
	*Protium*	*Protium heptaphyllum* (Aubl.)	Amescla	Native	TR	Sap/resin & Seeds	Toothache, Stomach problems	Sterile material
capparaceae	*Tarenaya*	*Tarenaya spinosa* (Jacq.) Raf.	Mussambe	Native	TR	Flower & Roots	Flu & colds	Sterile material
Euphorbiaceae	*Astraea*	*Astraea lobata* (L.) Klotzsch	Alfavaca de cobra	Native	TR	Roots	Stinging of venomous animals	Sterile material
	*Jatropha*	*Jatropha gossypiifolia* L.	Pinhão Roxo	Native	SDTF	Leaves, shoot, sap & resin	Stinging of venomous animals	IPA91702
Fabaceae	*Amburana*	*Amburana Cearensis* (Allemão) A.C. Sm.	Imburana de cheiro	Native	SDTF	Bark, Leaves & Seeds.	Diarrhea, Headache, Flu & colds, Indigestion & Cough	Sterile material
	*Anadenanthera*	*Anadenanthera colubrina* var. cebil (Griseb.) Altschul	Angico	Native	SDTF	Bark	Injury, Flu & colds & Inflammation in general	IPA91649
	*Bauhinia*	*Bauhinia acuruana* Moric.	Mororó	Native	SDTF	Bark, Leaves & Roots	Diabetes, Flu & colds, Inflammation in general & Cough	IPA91660
	*Copaifera*	*Copaifera* sp.	Pau D’óleo	Native	TR	Sap & Resin	Pain in general & stroke	Sterile material
	*Hymenaea*	*Hymenaea courbaril* L.	Jatobá	Native	SDTF	Bark, fruit & roots	Anemia, Expectorant, Injury, Stomach problems, Flu & colds, Airway inflammation, Inflammation in general & Cough	IPA91630
					TR	Bark & fruit	Injury & Airway inflammation	Sterile material
	*Libidibia*	*Libidibia ferrea* (Mart. ex Tul.) L.P. Queiroz	Jucá	Native	SDTF	Bark & fruit	Toothache	IPA91696
	*Mimosa*	*Mimosa tenuiflora* (Willd.) Poir.	Jurema Preta	Native	SDTF	Bark	Injury	Sterile material
	*Periandra*	*Periandra mediterranea* (Vell.) Taub.	Alcançu	Native	SDTF	Bark, Leaves & Roots	Expectorant, Flu & colds, Airways inflammation & Cough	IPA91648
	*Pityrocarpa*	*Pityrocarpa moniliformis* (Benth.) Luckow & R.W. Jobson	Canzenzo	Native	SDTF	Bark	Diarrhea	IPA91651
	*Poincianella*	*Poincianella microphylla* (Mart. ex G. Don) L.P. Queiroz	Catingueira rasteira	Native	SDTF	Bark, flower & Roots	Inflammation in general & Cough	IPA91653
	*Prosopis*	*Prosopis juliflora* (Sw.)	Algaroba	Exotic	SDTF	Bark	Inflammation in general	Sterile material
	*Senegalia*	*Senegalia bahiensis* (Benth.) Seigler & Ebinger	Carcará	Native	SDTF	Bark & Roots	Spine problems & Kidney problems	IPA91697
	*Senna*	*Senna occidentalis* (L.) Link	Mangerioba	Native	TR	Flower & Seeds	Headache, Airways Inflammation	IPA91706
	*Senna*	*Senna spectabilis* var. excelsa (Schrad.) H.S. Irwin & Barneby	Canafístula	Native	SDTF	Bark	Diarrhea	HST22166
Hypericaceae	*Vismia*	*Vismia guianensis* (Aubl.) Choisy	Lacre	Native	TR	Bark, Leaves & Roots	Arterial hypertension & Renal problems	IPA91717
Lamiaceae	*Lippia*	*Lippia origanoides* Kunth	Alecrim do Mato	Native	SDTF	Leaves	Toothache & Headache	IPA91612
	*Mentha*	*Mentha piperita* L.	Hortelã da folha pequena	Exotic	SDTF	Leaves & roots	Expectorant, Flu & colds, Airways Inflammation & Cough	Sterile material
	*Ocimum*	*Ocimum gratissimum* L.	Alfavaca	Exotic	TR	Leaves	Stomach problems & conjunctivitis	Sterile material
	*Plectranthus*	*Plectranthus amboinicus* (Lour.) Spreng.	Hortelã da folha grande	Exotic	SDTF	Leaves	Flu & colds & Cough	Sterile material
	*Rosmarinus*	*Rosmarinus officinalis* L.	Alecrim	Exotic	SDTF	Leaves	Headache, Flu & colds	Sterile material
Lauraceae	*Persea*	*Persea americana* Mill.	Abacate	Exotic	SDTF	Leaves	Renal problems	HST22158
Malvaceae	*Guazuma*	*Guazuma ulmifolia* Lam.	Mutamba	Native	TR	Bark	Bone fracture & Cough	IPA91718
Maranthaceae	*Maranta*	*Maranta* sp.	Uruba	Native	TR	Roots	Stinging of venomous animals	Sterile material
Moraceae	*Sorocea*	*Sorocea* sp.	Pau Teiu	Native	SDTF	Bark, sap & resin	Stinging of venomous animals.	Sterile material
Myrtaceae	*Plinia*	*Plinia cauliflora* (Mart.) Kausel	Jabuticaba	Native	SDTF	Bark	Diarrhea	Sterile material
	*Psidium*	*Psidium guajava* L.	Goiaba	Exotic	SDTF	Bark & Leaves	Diarrhea	Sterile material
	*Psidium*	*Psidium guineense* Sw.	Araçá	Native	TR	Leaves	Diarrhea	IPA91708
	*Psidium*	*Psidium* sp.	Araçá	Native	SDTF	Bark	Diarrhea	Sterile material
	*Syzygium*	*Syzygium cumini* (L.) Skeels	Azeitona Roxa	Exotic	TR	NA	Diabetes	Sterile material
Olacaceae	*Ximenia*	*Ximenia americana* linn	Ameixa	Native	SDTF	Bark & Leaves	Injury, Throat problems, Stomach problems, Inflammation in general & Gynecological problem	Sterile material
Passifloraceae	*Passiflora*	*Passiflora cincinnata* Mast.	Maracujá do Mato	Native	SDTF	Leaves, fruit, roots & Seeds.	Calming, Flu & colds, Inflammation in general & Cough	IPA91635
	*Passiflora*	*Passiflora edulis* Sims	Maracujá	Native	SDTF	Leaves	Indigestion	Sterile material
	*Passiflora*	*Passiflora foetida* L.	Maracujá de Estralo	Native	SDTF	Leaves	Flu & colds & Conjunctivitis	IPA91677
Phyllanthaceae	*Phyllanthus*	*Phyllanthus urinaria* L.	Quebra Pedra	Native	SDTF	Entire plant & Roots	Renal problems	Sterile material
					TR	Entire plant or Roots	Renal problems	Sterile material
Plumbaginaceae	*Plumbago*	*Plumbago scandens* L.	Louco	Native	SDTF	Stem, Leaves & Roots	Toothache	HST22163
Poaceae	*Cymbopogon*	*Cymbopogon citratus* (DC) Stapf.	Capim Santo	Exotic	SDTF	Leaves	Calming, Diarrhea, Flu & colds, Hypertension & Indigestion	Sterile material
					TR	Leaves	Diarrhea	Sterile material
Punicaceae	*Punica*	*Punica granatum* L.	Romã	Exotic	SDTF	Bark, Leaves, Fruit & Seeds	Throat problems, Stomach problems & Inflammation in general	Sterile material
Rhamnaceae	*Ziziphus*	*Ziziphus joazeiro* Mart.	Juazeiro	Native	TR	Bark	Expectorant, Toothache, Flu & colds, Airway inflammation & Cough	Sterile material
					SDTF	Bark, Leaves & Roots	Expectorant, Acariasis & other infestations & Cough	IPA91676
Rubiaceae	*Borreria*	*Borreria verticillata* (L.) G. Mey.	Vassoura de botão	Native	TR	Entire plant or Roots	Stroke	IPA91713
	*Genipa*	*Genipa americana* L.	Genipapo	Native	TR	Bark & fruit	Anemia	Sterile material
	*Tocoyena*	*Tocoyena formosa* (Cham. & Schltdl.) K. Schum.	Genipapo	Native	SDTF	Bark	Stroke	IPA91611
Rutaceae	*Ruta*	*Ruta graveolens* L.	Arruda	Exotic	SDTF	Leaves	Headache, Pain in general	Sterile material
Sapotaceae	*Sideroxylon*	*Sideroxylon obtusifolium* (Roem. & Schult.) T.D. Penn.	Quixabeira	Native	SDTF	Bark	Injury, General inflammation, Stroke & Gynecological problem	Sterile material
Solanaceae	*Solanum*	*Solanum paniculatum* L.	Jurubeba	Native	SDTF	Leaves, fruit, roots & Seeds	Injury, Stomach problems, Flu & colds, Inflammation in general & Cough	IPA91633
					TR	Bark, Flower, Leaves, Fruit, Entire Plant, Roots, Sap/Resin & Seeds	Expectorant, Flu & colds & Cough	IPA91709
Violaceae	*Pombalia*	*Pombalia arenaria* (Ule) Paula-Souza	Papaconha	Native	SDTF	Bark & Roots	Expectorant, Flu & colds, Airways Inflammation & Cough	IPA91628

**Note:**

* SDTF indicates that the species in question was obtained in the Seasonally Dry Tropical Forest, while TR indicates that the species was collected from the Tropical Rainforest.

### Data analysis

The identification of utility equivalents and utility redundancies between the Caatinga and Atlantic Forest areas was performed through a similarity analysis (Jaccard). A binary matrix was constructed by gathering data from the interviews, setting the plants as objects and the therapeutic indications as descriptors. When a plant was used for a given therapeutic indication, the cell was filled with value 1. When a plant was not used for a given therapeutic indication, the corresponding cell had the value zero. In relation to utilitarian equivalence, the analysis now considered the pairs formed by plants of the same species, in cases in which the same species occurred in both regions, sometimes not considered. When considering pairs of the same species, the objective was to verify if plants that include the same biological entity, and that naturally share common traits, contribute to a scenario of overlapping medicinal uses among taxonomically close plants.

Still, when the same botanical species was mentioned in the two regions, it entered the matrix as two distinct entities (Plant A—Atlantic Forest and Plant A—Caatinga). Based on the binary matrix, the Jaccard similarity matrix was constructed. Pairs of plants with more than 50% of similarity were registered and classified as “redundant” (when they were plants from the same region) and “equivalents” (when they were plants from different regions).

For the analysis of the influence of taxonomic affiliation in favor of the establishment of utilitarian equivalence and utilitarian redundancy among the botanical species, the odds ratio (OR) was used, which is more indicated when there are small values, since the amount of pairs was much lower than the number of non-equivalent pairs. The OR tested:

(1) if pairs formed by plants of the same genus are more likely to be equivalent; (2) if pairs formed by plants of the same family are more likely to be equivalent; (3) if pairs formed by plants of the same genus are more likely to be redundant; (4) if pairs formed by plants of the same family are more likely to be redundant.

The *p* value for each test was calculated by testing the null hypothesis of independence between the genus/family variables and the utilitarian equivalence. The same approach was used to test the relationship between genus/family variables and utilitarian redundancy. The odds ratio calculations were performed by the oddsratio function of the fmsb package available in the statistical program R, version 3.2.2 (The R Foundation for Statistical Computing). For the processed tests, *p* < 0.05 was considered.

## Results

### Analysis of the influence of the taxonomic affiliation in favor of the establishment of utilitarian equivalence and utilitarian redundancy

The species cited by more than one local expert in each area are shown in Table 2. The results of the analyzes that did not consider pairs of the same species (Table 3) showed that the chances of a pair being utilitarian equivalent increased when they were species of the same genus (OR = 9.25, CI [1.68–51.02], *p* < 0.05). The family variable was not significant, although the *p* value was close to 0.05 (OR = 1.87, CI [0.92–3.80], *p* = 0.08). However, when the analyzes also considered the pairs formed by the same botanical species mentioned in both regions (Table 4), the chances of pairs of the same family being equivalent increased a lot. The family variable was significant (OR = 2.7, CI [1.49–4.88], *p* < 0.001) and the genus variable also remained significant, however with an even higher OR value (OR = 11.77, CI [4.48–30.93]; *p* < 0.0001). Although this last finding does not fit the concept of utilitarian equivalence, because it is the same taxonomic entity, it supports the idea that plants used for the same ends share common traits. Following this rationale, we also chose to discuss this finding. These results confirm the hypothesis that there is influence of the taxonomic affiliation on the establishment of utilitarian equivalence (see Table 4).

**Table 3:** Equivalence without pairs of the same species: results of the odds ratio test that verified whether pairs formed by plants of the same genus or of the same family are more likely to be utilitarian equivalents.

Variables* (*n* = 2,268)	Total	Equivalents	%	OR	IC 95%	*p*
**Genus**						
Equal	6	2	33.3	9.25	[1.68–51.02]	<0.01
Different	2,262	116	5.1			
**Family**						
Equal	100	9	9.0	1.87	[0.92–3.80]	0.0804
Different	2,168	109	5.0			

**Note:**

* The variables are organized as: total pairs of the same genus; equivalent pairs of the same genus; total pairs of different genera; equivalents of different genera; total pairs of the same family; pairs equivalents of the same family; total pairs of different families; equivalents of different families. In the OR column, the values of association between the variables (genus and family) and equivalence are indicated; In column % the proportion of pairs that are and that are not formed by species of the same genus and family are indicated; In the IC column, the confidence intervals for OR values are indicated; In column *p*, the values of significance are indicated, values <0.05 being indicative that the results obtained were not by chance.

**Table 4:** Equivalence with pairs of the same species: results of the odds ratio test that verified whether pairs formed by plants of the same genus or of the same family are more likely to be utilitarian equivalents.

Variables* (*n* = 2,268)	Total	Equivalents	%	OR	IC 95%	*p*
**Genus**						
Equal	18	7	38.9	11.77	[4.48–30.93]	<0.0001
Different	2,262	116	5.1			
**Family**						
Equal	112	14	12.5	2.7	[1.49–4.88]	<0.001
Different	2,168	109	5.0			

**Note:**

* The variables are organized as: total pairs of the same genus; equivalent pairs of the same genus; total pairs of different genera; equivalents of different genera; total pairs of the same family; pairs equivalents of the same family; total pairs of different families; equivalents of different families. In the OR column, the values of association between the variables (genus and family) and equivalence are indicated; In column % the proportion of pairs that are and that are not formed by species of the same genus and family are indicated; In the IC column, the confidence intervals for OR values are indicated; In column *p*, the values of significance are indicated, values <0.05 being indicative that the results obtained were not by chance.

In the case of utilitarian redundancy, there was no positive correlation between the genus variable and the Redundancy establishment (OR = 2.21, CI [0.27–17.79], *p* = 0.44). On the other hand, the chances of a pair being redundant increased when they were species of the same family (OR = 1.94, CI [1.06–3.53], *p* < 0.05). These results confirm the hypothesis that there is influence of the taxonomic affiliation, in terms of family, on the establishment of utilitarian redundancy (see Table 5).

**Table 5:** Results of the odds ratio test that verified whether pairs formed by plants of the same genus or the same family are more likely to be utilitarian redundants.

Variables* (*n* = 2,473)	Total	Redundants	%	OR	IC 95%	*p*
**Genus**						
Equal	9	1	11.1	2.21	0.27-17.79	0.4451
Different	2,464	132	5.4			
**Family**						
Equal	137	13	9.5	1.94	1.06-3-53	<0.05
Different	2,336	120	5.1			

**Note:**

* The variables are organized as: total pairs of the same genus; equivalent pairs of the same genus; total pairs of different genera; equivalents of different genera; total pairs of the same family; pairs equivalents of the same family; total pairs of different families; equivalents of different families. In the OR column, the values of association between the variables (genus and family) and equivalence are indicated; In column % the proportion of pairs that are and that are not formed by species of the same genus and family are indicated; In the IC column, the confidence intervals for OR values are indicated; In column *p*, the values of significance are indicated, values <0.05 being indicative that the results obtained were not by chance.

## Discussion

### There are more chances of there being utilitarian equivalence among pairs that include species of the same genus

The prediction that local experts from the different regions, Atlantic Forest and Caatinga, have great chances of selecting the same genus or the same botanical family to treat similar diseases was confirmed, indicating that knowledge production in plant-based medical systems is influenced by intrinsic characteristics of the botanical species. There are indications in other countries that the selection of medicinal plants suffers this type of taxonomic influence. Saslis-Lagoudakis et al. (2012), for example, analyzed the medicinal flora of three distinct regions, Cape region (South Africa), Nepal and New Zealand, and found a close phylogenetic proximity between species used to treat similar diseases.

In addition, worthy of note is the study by Molander et al. (2011), which addressed the treatment of venomous snake bites and analyzed the local medicinal flora of Brazil, Nicaragua, Nepal, China and South Africa. As a result, families Apocynaceae, Lamiaceae and Rubiaceae performed as overused groups in at least two of the five countries. In addition, analyzes at the genus level have shown that the Piper L. group was overused in at least two of the countries described (Chase et al., 2016).

The obvious patterns in terms of convergence of medicinal uses among taxonomically close plants are probably related to the sharing of secondary compounds among species of the same taxonomic group (Molander et al., 2011). In a study comparing the botanical genera of Peru and Mali, Bletter (2006) found that the genera present in both countries were used for the same medicinal purposes. Since it is difficult to disseminate knowledge among the peoples of the two regions, given the geographic distance and the absence of historical relations between the two areas, it is more likely that the different peoples have reached the same conclusions about the use of plants independently, that is, evolutionary convergence (Bletter, 2006).

If, on the one hand, the chemical efficacy provided by the chemical repertoire of each botanical family can explain the utilitarian equivalence among taxonomically close plants, this does not exclude the possibility that, processes of knowledge transmission among peoples of different regions may have contributed to the formation of the observed scenario. If we consider that 14 out of the 64 species analyzed are not native to Brazil (12 in the FTSS and 5 in the FTU), with most of these exotic species coming from the Old World, it is probable that the diffusion of knowledge of use is among the factors underlying the incorporation of these plants into local medical systems.

Some local medical systems in Northeast Brazil also carry a strong influence of African cultural matrices, due to a regime of slavery that brought Africans to the country, but especially to that region (Albuquerque, 2014). It should be noted that Albuquerque (2011) investigated cases of substitution of medicinal species of the same genus, *Ocimum* L. (Lamiaceae), carried out by Africans when they arrived in Brazil, and concluded that the substitution process obeyed morphological similarities, namely plant, type of inflorescence and fruit (Albuquerque, 2011). In addition, pharmacological studies demonstrate that many of these substitute species have the same biological activity observed for African medicinal species of this genus (Albuquerque, 2011). Therefore, utilitarian equivalence cases may be related to both historical knowledge of a past social group and chemical similarities between equivalent plants.

### Utilitarian redundancy between two species is associated with their taxonomic affiliations

Our findings also demonstrated that the utilitarian redundancy among botanical species may be associated with their taxonomic affiliations in terms of family. As discussed in the case of equivalence, taxonomic proximity may imply the sharing of other traits such as the presence of certain classes of chemical compounds (Rønsted et al., 2012) and thus utilitarian redundancy may also emerge from the similarity of the chemical repertoires of certain plants. Some studies have inferred on the chemical influence on the configuration of redundancy scenarios. In a study in the Caatinga area, Santoro (2014) dealt with the factors underlying the process of choosing and incorporating medicinal plants with the same therapeutic indications and found that most of the diseases considered to be more serious are not very redundant (they have few plants that can treat them). One explanation for this scenario is that more serious diseases may require more specific treatments, requiring chemical compounds restricted to certain plants, whereas less serious diseases allow treatment from a wider spectrum of chemical compounds found in several plants (Santoro, 2014; Medeiros & Albuquerque, 2015).

Although Santoro (2014) have not performed chemical analyzes to demonstrate this interpretation, there is evidence of the importance of phylogeny for character distribution responsible for certain pharmacological activities. Rønsted et al. (2012), for example, have shown a correlation between phylogenetic proximity and the diversity and inhibitory activity of alkaloids to the enzyme acetylcholinesterase (AChE). In this sense, the selection of redundant plants from the same family would be partly explained by the sharing of an evolutionary past that culminated in similarities in terms of biosynthetic pathways responsible for secreting compounds of medicinal value (Rønsted et al., 2012).

Another way to approach the influence of taxonomic affiliation on the utilitarian redundancy scenario is that the local population of each region studied may be selecting medicinal plants based on certain cultural traits and local perceptions of their own. Knowing that plants of the same family tend to share the same classes of chemical compounds (Bletter, 2006), and that these compounds are responsible for the organoleptic characteristics (Casagrande, 2000), it is possible that flavor and odor characteristics serve as clues for selection of species for the treatment of certain diseases. Thus, the Arecaceae family, widely represented in the medicinal repertoire of several peoples (Moerman, 1991), may be selected based on its bitter taste, since it has a great variety of sesquiterpenes and other bioactive compounds, whose taste is bitter (Casagrande, 2000). Thus, cultural factors should also be considered for a broader understanding of the taxonomic influence on utilitarian redundancy.

### Limitations and prospects

One of the limitations of this study is the dynamic nature of the taxonomic classifications that guided our statistical analyzes. Considering the changes in the taxonomic groupings over the decades, which will undoubtedly still occur to some extent in the coming years, we consider that the conclusions regarding our model need to be minimally relativized. However, there are arguments in favor of using the family and genus categories in studies of this type. According to Evert & Eichhorn (2013), the association between molecular systematics and the study of morphological characters has brought greater reliability to the use of these taxonomic categories. The authors also state that almost all families of flowering plants currently occupy a well-supported phylogenetic position, and this is progressing well at the genus level. Besides, even the oldest studies, based on outdated classifications (based on external similarities), allowed relevant advances in terms of medicinal plant use patterns (Moerman, 1991).

It is also worth explaining that although we conducted this study in four communities, our small sample size may have influenced results. Our decision to consult only local specialists in medicinal plants, with a non-probabilistic sampling, and to exclude idiosyncratic information, led to a considerable reduction in the sample size for both interviewees and plants. Regarding the limited number of informants, our results may not be generalized for the whole communities but rather understood as a product of local experts’ knowledge and behaviors.

Concerning our limited number of species, since the main objective of this study is to recognize use patterns, we believe that adding idiosyncratic knowledge to the analysis could produce “noises” in the database and, consequently, decrease our explanatory power. Since we are dealing with tendencies, individual information would not be useful to our research design. However, future studies would gain by increasing the number of communities and (consequently) interviewees and plant species, making statistical procedures even more reliable.

Additionally, communities placed in the semi-arid region are established on the region longer than the TR populations, which may also have influenced the results (e.g., perhaps longer living in contact with the environment favored greater medical knowledge on native flora to treat a larger set of diseases, which could contribute to a more significant number of equivalent pairs). Therefore, we propose that future studies on utilitarian equivalence include TR communities with longer histories in the region.

## Conclusions

Here, both utilitarian equivalence and utilitarian redundancy have been taxonomically influenced, possibly due to the chemical similarities that taxonomically close plants tend to possess and due to cultural traits that modulate plant selection and the transmission of knowledge. However, the central element behind the taxonomic influence seems to be the therapeutic efficacy, that is, the success of the plant in curing a particular disease, which in turn is strongly related to the repertoire of chemical compounds of the species. In addition, the utilitarian equivalence approach between local medical systems of dry forests and wetlands has shown that even among communities that are subject to characteristic climates and distinct native floras, there are similar adaptive responses. In this sense, the mechanisms of selection of medicinal plants involve certain behaviors and perceptions common to different peoples. Regarding the choice of plants for pharmacological studies, our findings indicate the feasibility of using a chemosystematic approach, that is, selection of plants phylogenetically close to the species with previously proven pharmacological activity.

## Supplemental Information

10.7717/peerj.9664/supp-1 Supplemental Information 1 Semistructured interview form.

10.7717/peerj.9664/supp-2 Supplemental Information 2 Raw data. Digitized information obtained through questionnaires. The data consists of information concerning the plants identified by each informant and their therapeutic indications.

## Additional Information and Declarations

### Field Study Permissions

The following information was supplied relating to field study approvals (i.e., approving body and any reference numbers): An authorization for scientific activities in the Catimbau National Park area (PARNA Catimbau) was given by Instituto Chico Mendes de Conservação da Biodiversidade (ICMBio/SISBIO), an agency linked to the Brazilian Ministry of the Environment (MMA), for the collection of botanical material (authorization number 67801-1).

### Data Availability

The following information was supplied regarding data availability: The raw measurements are available in a Supplemental File.
