# Supplementary material for: Taxonomic affiliation influences the selection of medicinal plants among people from semi-arid and humid regions—a proposition for the evaluation of utilitarian equivalence in Northeast Brazil
Source: PeerJ. 2020 Aug 4;8:e9664. doi: 10.7717/peerj.9664 (PMC7413083; doi:10.7717/peerj.9664)
Supplement: Supplemental Information 1 [file peerj-08-9664-s001.docx]

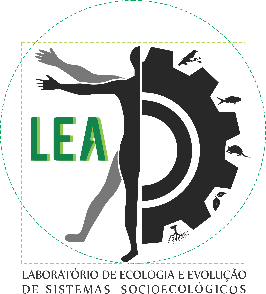


DATA COLLECTION ABOUT LOCAL KNOWLEDGE OF MEDICINAL PLANTS
(INTERVIEW FORM)

Interview number ______

Name:

Nickname:

Community:

1. Which medicinal plants do you know?

2. What health problems is this plant useful for?

3. What part of the plant is used?

4. How is this medicinal plant prepared for consumption?

5. Does this plant have any flavor? Which one? Is this taste good, bad, or neither good nor bad?
